# Supplementary material for: Time‑resolved multi-omic analysis of paclitaxel exposure in human iPSC‑derived sensory neurons unveils mechanisms of chemotherapy‑induced peripheral neuropathy
Source: Cell Death Dis. 2026 Feb 10;17(1):211. doi: 10.1038/s41419-026-08445-2 (PMC12921266; doi:10.1038/s41419-026-08445-2)
Supplement: Supplementary file 15 — Dataset 1. [file 41419_2026_8445_MOESM15_ESM.zip › CDD_Open_Data/04_RNA_Seq/Timeline_panel_plots_etc_2023-01-23_1609.zip!/Panel_Plots_etc/Evidence_Plots/72h/evidence_plot_standard contrasts_msigdb_reactome_72h_PTX_vs_DMSO_ID0_M27067_REACTOME_TRANSPORT_OF_CONNEXONS_TO_THE_PLASMA_MEMBRANE.pdf]

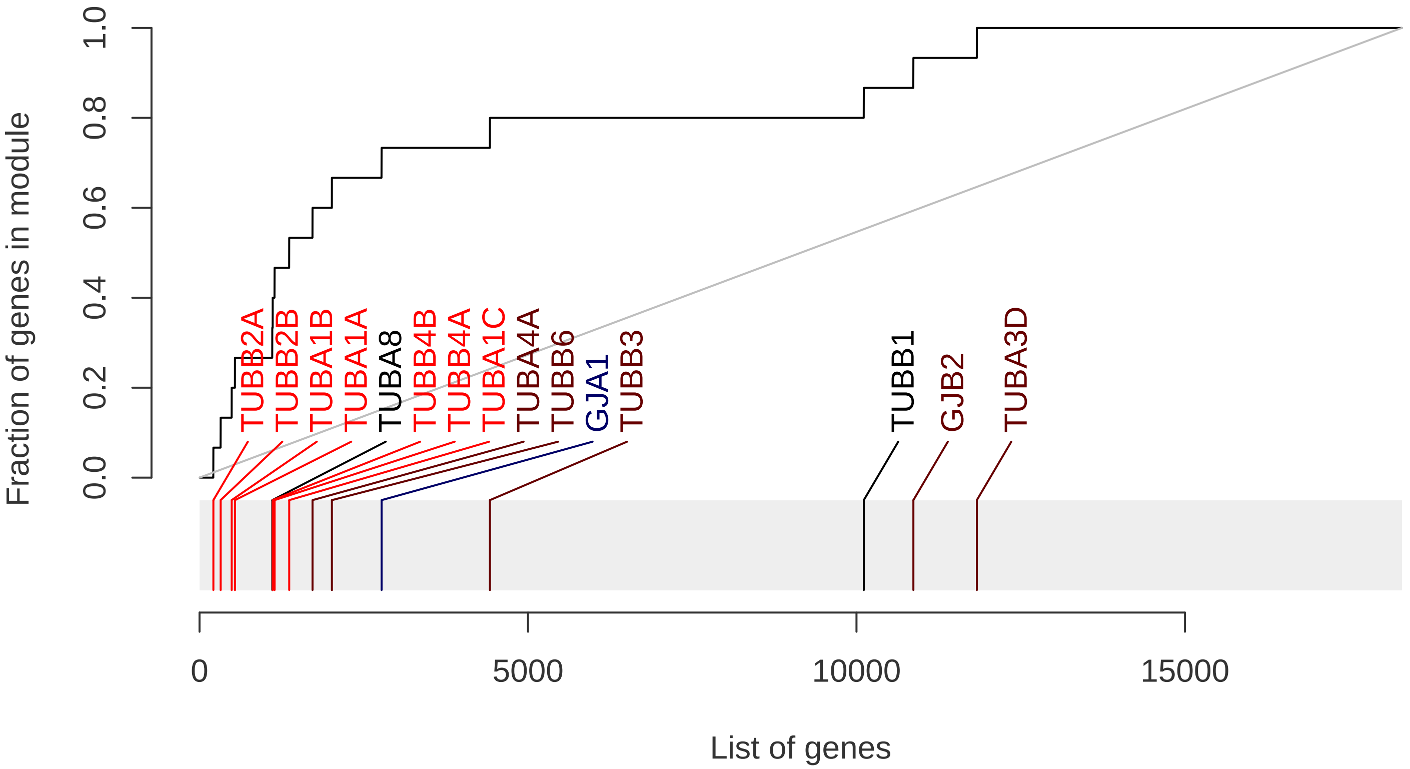
Fraction of genes in module
0.0
0.2
0.4
0.6
0.8
1.0
TUBB2A
TUBB2B
TUBA1B
TUBA1A
TUBA8
TUBB4B
TUBB4A
TUBA1C
TUBA4A
TUBB6
GJA1
TUBB3
TUBB1
GJB2
TUBA3D
0
5000
10000
15000
List of genes
